# Red Cell Distribution Width and RDW-to-Platelet Ratio Patterns Across the Spectrum of Hypoxic–Ischemic Encephalopathy

**DOI:** 10.3390/children13010100

**Published:** 2026-01-10

**Authors:** Seray Öztürk, Gülsüm Kadıoğlu Şimşek, Burak Özdemir, Mahmut Mert Ercel, Betül Siyah Bilgin, Hayriye Gözde Kanmaz Kutman

**Affiliations:** 1 Department of Neonatology, Ankara Bilkent City Hospital, 06800 Ankara, Turkey; seray.ozturk2@saglik.gov.tr; 2 Department of Neonatology, University of Health Sciences, Ankara Bilkent City Hospital, 06800 Ankara, Turkey; betul.siyahbilgin@sbu.edu.tr (B.S.B.); hayriyegozde.kanmazkutman@sbu.edu.tr (H.G.K.K.); 3 Department of Pediatrics, Ankara Bilkent City Hospital, 06800 Ankara, Turkey; burak.ozdemir@saglik.gov.tr (B.Ö.); mahmutmert.ercel@saglik.gov.tr (M.M.E.)

**Keywords:** therapeutic hypothermia, red cell distribution width, RDW-to-platelet ratio, inflammation

## Abstract

**Highlights:**

**What are the main findings?**

- Across the spectrum of hypoxic–ischemic encephalopathy, RDW demonstrated a blunted postnatal decline, while the RDW-to-platelet ratio (RPR) showed a mild, non-significant increase during the early neonatal period, with higher RPR levels observed in association with increasing HIE severity across Sarnat stages.
- RDW values decreased significantly in infants with mild HIE, reflecting preserved postnatal hematologic adaptation, whereas RDW remained relatively stable in infants with moderate-to-severe HIE receiving standard care treatment.

**What is the implications of the main finding?**

- RDW and RPR, as simple and routinely accessible hematologic indices, may provide complementary information on early inflammatory and hematologic responses in HIE cases. These parameters should be interpreted as descriptive markers of hematologic trajectories rather than as indicators guiding clinical intervention.

**Abstract:**

**Background:** Red cell distribution width (RDW) and the RDW-to-platelet ratio (RPR) have emerged as readily available hematologic markers reflecting systemic inflammation in neonates with hypoxic–ischemic encephalopathy (HIE); however, their early postnatal trajectories across the clinical spectrum of HIE remain insufficiently characterized. **Methods:** This retrospective cohort study included 229 term or near-term infants diagnosed with HIE. Among them, 166 infants received therapeutic hypothermia, whereas 63 infants who did not undergo cooling served as the reference group. RDW and RPR values were measured at birth and at 72 h of life (after completion of cooling in the hypothermia group). **Results:** In the reference group, RDW values significantly decreased at 72 h, reflecting normal postnatal hematologic adaptation. In contrast, the hypothermia group demonstrated a blunted decline, with RDW levels remaining relatively stable over the first 72 h, consistent with a blunted early postnatal RDW decline. RPR values showed a mild, non-significant upward trend during the first 72 h of life; however, exploratory analyses suggested an association between higher RPR levels and increasing HIE severity. **Conclusions:** Across the spectrum of hypoxic–ischemic encephalopathy, RDW demonstrated a blunted postnatal decline, whereas RPR showed a mild, non-significant increase during the early neonatal period. These readily available hematologic markers may provide complementary insights into early systemic inflammatory and hematologic responses in HIE. Prospective multicenter studies are needed to determine their prognostic value and relationship with clinical and neurodevelopmental outcomes.

## 1. Introduction

Hypoxic–ischemic encephalopathy (HIE) is a major cause of neonatal mortality and long-term neurodevelopmental impairment, with an incidence of approximately 1–8 per 1000 live births in developed countries [1]. Therapeutic hypothermia is the standard treatment for infants with moderate-to-severe HIE and has been shown to significantly reduce mortality and severe neurological sequelae [2]. Beyond its neuroprotective effects, HIE triggers a complex systemic inflammatory response driven by hypoxia and subsequent reoxygenation injuries [1]. Accordingly, complete blood count–derived inflammatory indices may be altered in affected infants. Previous studies have shown that markers such as the neutrophil-to-lymphocyte ratio (NLR) and platelet-to-lymphocyte ratio (PLR) are significantly higher in moderate and severe HIE than in healthy newborns, and that these indices decrease following therapeutic hypothermia [3]. These findings reflect the presence of systemic inflammation, hematopoietic stress, and bone marrow responses in HIE.

Red cell distribution width (RDW), an indicator of erythrocyte volume variability, is frequently elevated in anemia, inflammation, and critical illnesses. Higher RDW values have been associated with adverse outcomes in adult and pediatric intensive care settings, and in neonatal sepsis, an RDW above 17–18% has been identified as an independent predictor of mortality [4]. The RDW/platelet ratio (RPR), recently proposed as an additional inflammatory marker, has been shown to correlate with SNAP-II scores and may help predict NICU morbidity and mortality [5,6,7]. Despite the growing interest, limited data exist regarding the behavior of RDW and RPR in neonates with HIE. Perinatal asphyxia–related tissue hypoxia and intensive medical interventions applied when necessary (e.g., fluids or transfusions) may influence erythrocyte indices. Additionally, both perinatal asphyxia and therapeutic hypothermia can contribute to thrombocytopenia and alterations in several hematologic parameters [8,9].

Ethical constraints prevented the formation of a control group consisting of moderate or severe HIE infants who did not receive therapeutic hypothermia. Infants with mild HIE (Sarnat Stage I) were used as the reference group, as withholding therapeutic hypothermia from eligible infants is ethically unacceptable. Although mild HIE represents a less severe form of hypoxic injury, it remains part of the HIE spectrum and reflects exposure to perinatal hypoxia, making this group a clinically relevant reference group rather than a physiologically equivalent control group. In this study, we describe the early trajectories of RDW and the RDW-to-platelet ratio across the clinical spectrum of hypoxic–ischemic encephalopathy, comparing infants receiving standard-of-care therapeutic hypothermia with a clinically relevant mild HIE reference group. By examining hematologic trajectories across the clinical spectrum of HIE severity, this study aimed to contribute to the limited literature on hematologic alterations accompanying hypoxia-ischemia and its treatment.

## 2. Materials and Methods

This retrospective cohort study reviewed the medical records of neonates diagnosed with HIE who were admitted to the neonatal intensive care unit between 2020 and 2023. The inclusion criteria were as follows: gestational age ≥36 weeks, diagnosis of HIE within the first 6 h after birth (according to clinical and neurological findings and Sarnat staging), and evaluation of the indication for therapeutic hypothermia in appropriate cases. A whole-body cooling protocol was applied for 72 h in infants with moderate (Sarnat Stage II) or severe (Stage III) HIE. Infants diagnosed with mild HIE (Stage I) or those who did not receive hypothermia treatment for any reason were considered the reference group. The study included newborns with complete blood count values available at birth (before hypothermia) and who received hypothermia treatment (hypothermia group), as well as infants diagnosed with HIE who did not receive treatment (reference group) during the same period. Cases for which a laboratory check at the 72nd hour could not be performed due to early exitus during follow-up were excluded from the study, as post-treatment RDW and platelet measurements could not be obtained, resulting in incomplete longitudinal data. Early onset sepsis (EOS) was defined as culture-proven or clinically suspected sepsis occurring within the first 72 h of life. Late-onset sepsis (LOS) was defined as sepsis diagnosed after 72 h of age, confirmed by clinical and/or microbiological evidence. Oliguria was defined as a urine output of < 1 mL/kg/h for at least 24 h. Seizure diagnosis was based on clinical observation and/or electroencephalography. Discharge was defined as survival to hospital discharge after the completion of clinical follow-up.

Standard Management of HIE: In our unit, the diagnosis and management of HIE follow a standardized algorithm based on the recommendations of the Turkish Neonatal Society Guidelines for Neonatal Encephalopathy [10]. All infants with suspected HIE in the delivery room were evaluated using cord blood gas analysis and a detailed physical examination after appropriate resuscitation. Infants diagnosed with HIE were admitted to the neonatal intensive care unit, where their vital signs, urine output, and amplitude-integrated EEG (aEEG) were continuously monitored. The severity of HIE was determined using the Sarnat classification. Therapeutic hypothermia was initiated within the first 6 h of birth for infants classified as Sarnat Stage II or III. Whole-body cooling was performed using TecoTherm™ total body cooling system (Inspiration Healthcare Ltd., Croydon, UK; manufactured in Germany) devices, maintaining the core temperature at 33–34 °C for 72 h. Rewarming was conducted under device control at a rate of 0.5–1 °C/h. During hospitalization, laboratory evaluations included blood gas analysis, complete blood count (CBC), blood cultures, and serum glucose level measurements. Within the first six postnatal hours, acute-phase reactants, urea, creatinine, sodium, potassium, calcium, magnesium, uric acid, troponin I, CK-MB, lactate dehydrogenase, liver enzymes, and coagulation parameters were obtained. Cranial ultrasonography and echocardiography were performed within the first 72 h as early as clinically feasible. Fluid restriction, inotropic support, and allopurinol therapy were initiated when clinically indicated. Empirical antibiotic therapy was initiated based on the infant’s clinical condition and acute-phase reactants.

Data Collection: Demographic data, clinical characteristics, and laboratory results were retrieved from patient records and electronic medical databases. RDW and platelet (PLT) values were obtained from the first blood sample collected at birth or upon admission to our unit and from the sample collected at the completion of the 72 h cooling period in the hypothermia group. In the reference group, CBC analyses were performed at birth and 72 h of life.

RDW was reported as “Red Cell Distribution Width (Coefficient of Variation)” in percentage (%). The RPR value was calculated by dividing the RDW percentage by the platelet count (in 10^9^/L). For example, an RDW of 18% and a PLT count of 200 × 10^9^/L yielded an RPR of 0.09. The primary outcomes of the study were the changes in RDW and RPR values before and after hypothermia treatment and the statistical significance of these changes. The secondary outcome was the comparison of these changes with those observed in the reference group.

Statistical Analysis: Data were analyzed using IBM SPSS Statistics 26.0 software. Continuous variables with normal distribution are presented as mean ± standard deviation, and categorical variables are presented as *n* (%). For comparisons between groups, Student’s *t*-test or the Mann–Whitney U test was used; for within-group comparisons (Days 1–4), the paired *t*-test or Wilcoxon signed-rank test was applied. For categorical variables, the chi-square or Fisher’s exact test was used. In all tests, a *p*-value of <0.05 was considered statistically significant. Additional exploratory multivariable analyses were performed using linear regression models with variance-robust estimation to account for potential heteroskedasticity. Additional exploratory analyses are provided in the Supplementary Material.

This study was conducted in accordance with the Declaration of Helsinki and approved by the Local Ethics Committee of the University of Health Sciences, Ankara Bilkent City Hospital (E2-22-1395, 16 February 2022).

## 3. Results

The hypothermia group consisted of 166 infants (Stage II: *n* = 142, Stage III: *n* = 21, and Stage I: *n* = 3), while the reference group included 63 infants (all with Stage I HIE). The demographic characteristics of the infants with and without hypothermia treatment are presented in Table 1.

### 3.1. Erythrocyte Parameters (Figure 1 and Figure 2, Table 2, Table 3 and Table 4)

**Figure 1:**
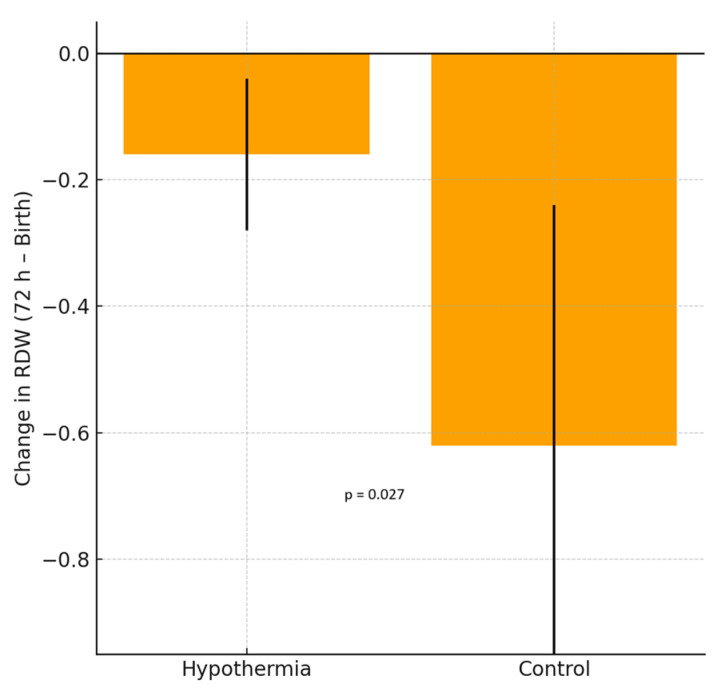
Change in red cell distribution width (RDW) between birth and 72 h in neonates with hypoxic–ischemic encephalopathy (HIE). The hypothermia group showed no significant difference, whereas the reference group demonstrated a significant decrease in RDW values. Bars represent the mean ± standard deviation (SD).

**Figure 2:**
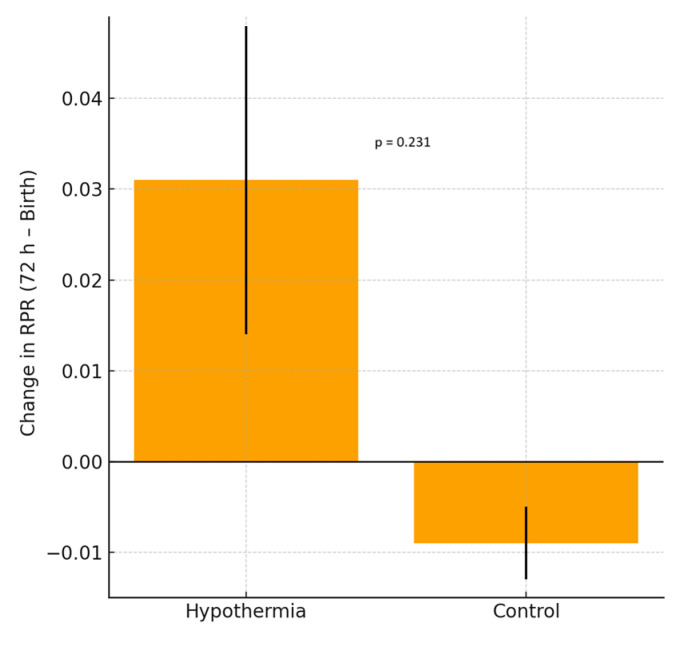
Change in the red cell distribution width-to-platelet ratio (RPR) between birth and 72 h in neonates with HIE. The hypothermia group demonstrated a mild, non-significant increase in RPR, whereas the reference group showed a slight, non-significant decrease in RPR. Bars represent the mean ± SD.

Among the erythrocyte indices, hemoglobin (HGB), mean corpuscular volume (MCV), mean corpuscular hemoglobin (MCH), and mean corpuscular hemoglobin concentration (MCHC) showed significant intragroup decreases in both the hypothermia and reference groups (all *p* < 0.001), consistent with the expected postnatal hemodilution. However, the Δ differences between the groups were not statistically significant. RDW values showed no statistically significant change in the hypothermia group (*p* = 0.055), indicating a trend toward stability rather than a definitive within-group effect, whereas a significant decline was observed in the reference group (*p* = 0.008). The between-group Δ difference was statistically significant (*p* = 0.027), indicating a blunted postnatal RDW reduction under hypothermia. RPR demonstrated a nonsignificant upward trend in the hypothermia group (*p* = 0.070). Nucleated red blood cell (NRBC) counts decreased markedly in both groups (*p* < 0.001), with a significantly greater reduction in the hypothermia group (Δ = −1.63 vs. −0.85, *p* = 0.045). As illustrated in Figure 1, RDW values significantly decreased between birth and 72 h in the reference group, reflecting normal postnatal hematologic adaptation. In contrast, the hypothermia group demonstrated no significant change in RDW during the same period, supporting a blunted decline under cooling treatment. As shown in Figure 2, the RDW-to-platelet ratio exhibited a divergent pattern: it increased slightly in the hypothermia group, whereas a mild decrease was noted in the mild HIE reference group. However, neither the within-group changes nor the between-group Δ comparison reached statistical significance. Overall, these findings indicate differences in early hematologic trajectories between infants treated with standard-of-care hypothermia and those in the mild HIE reference group. When hematologic parameters were further analyzed according to hypoxic–ischemic encephalopathy severity, stratified by Sarnat stage (I–III), both RDW and RDW-to-platelet ratio (RPR) values differed across severity groups. RDW values at birth and at 72 h were higher with increasing HIE severity, whereas changes in RDW over time did not significantly differ across stages. In contrast, RPR values at birth, at 72 h, and their postnatal changes showed a stepwise increase with increasing HIE severity (Table 3). In exploratory multivariable linear regression analyses adjusting for clinically relevant covariates, HIE severity was not independently associated with changes in RDW over the first 72 h. However, higher RPR values at 72 h were independently associated with moderate HIE (Sarnat stage II) compared with mild HIE (Sarnat stage I). No independent association was observed for severe HIE, likely reflecting limited statistical power due to smaller sample size (Table 4). Additional exploratory analyses evaluating postnatal changes in RDW and RPR, as well as analyses restricted to infants receiving therapeutic hypothermia, are presented in the Supplementary Material (Tables S1 and S2).

**Table 2:** Effect of hypothermia on hematologic parameters.

Parameter	Hypothermia 1st Measurement	Hypothermia 2nd Measurement	*p* (Within H)	Mild HIE Reference Group 1st Measurement	Mild HIE Reference Group 2nd Measurement	*p* (Within C)	Δ (H)	Δ (R)	*p* (H vs. R; Δ)
HGB (g/dL)	17.07 ± 2.44	14.49 ± 3.00	<0.001	17.05 ± 1.79	14.82 ± 2.91	<0.001	−2.58	−2.23	0.504
MCH (pg)	35.38 ± 1.48	34.71 ± 1.89	<0.001	34.82 ± 1.58	34.10 ± 1.87	0.014	−0.68	−0.72	0.891
MCHC (g/dL)	32.27 ± 1.40	33.40 ± 1.19	<0.001	32.49 ± 1.38	33.27 ± 1.18	0.050	1.13	0.78	0.365
MCV (fL)	112.32 ± 6.54	103.90 ± 9.54	<0.001	110.54 ± 5.30	100.62 ± 10.60	<0.001	−8.42	−9.91	0.353
RDW (%)	17.28 ± 1.37	17.11 ± 1.73	0.055	17.09 ± 1.33	16.46 ± 1.78	0.008	−0.17	−0.62	0.027
RDW/PLT	0.10 ± 0.13	0.13 ± 0.21	0.070	0.07 ± 0.03	0.06 ± 0.03	0.081	0.03	−0.01	0.231
WBC (×10^3^/µL)	19.99 ± 6.98	10.24 ± 4.88	<0.001	16.73 ± 6.10	9.97 ± 4.36	<0.001	−9.75	−6.76	0.029
NEU (×10^3^/µL)	11.32 ± 5.78	5.59 ± 3.80	<0.001	10.72 ± 5.02	4.73 ± 2.88	<0.001	−5.73	−5.99	0.832
LYM (×10^3^/µL)	5.62 ± 3.73	3.16 ± 1.51	<0.001	4.20 ± 1.75	3.41 ± 1.20	0.023	−2.46	−0.79	0.031
MONO (×10^3^/µL)	1.08 ± 0.55	0.77 ± 0.51	<0.001	1.08 ± 0.52	1.11 ± 0.58	0.761	−0.31	0.03	0.006
BASO (×10^3^/µL)	0.22 ± 0.30	0.06 ± 0.11	<0.001	0.13 ± 0.10	0.06 ± 0.06	<0.001	−0.15	−0.07	0.076
NRBC (/100 WBC)	2.03 ± 2.37	0.40 ± 0.66	<0.001	1.06 ± 1.17	0.21 ± 0.26	<0.001	−1.63	−0.85	0.045
DNI (%)	4.48 ± 5.90	5.21 ± 7.90	0.503	2.42 ± 1.38	2.78 ± 2.50	0.740	0.73	0.36	0.900
LUC (×10^3^/µL)	0.57 ± 0.93	0.29 ± 0.23	<0.001	0.26 ± 0.22	0.31 ± 0.19	0.227	−0.27	0.05	0.027
PLT (×10^3^/µL)	254.00 ± 91.57	251.97 ± 141.19	0.830	289.32 ± 100.20	343.46 ± 151.57	0.004	−2.03	54.15	0.007
MPV (fL)	8.98 ± 0.99	9.16 ± 1.23	0.074	8.88 ± 0.98	8.96 ± 1.00	0.698	0.18	0.08	0.635

Comparison of hematologic parameters between neonates treated with and without hypothermia. Values are presented as the mean ± SD. Within-group comparisons were performed using the paired *t*-test or Wilcoxon test, and between-group Δ comparisons were made using the independent *t*-test or Mann–Whitney U test. Statistical significance was set at *p* < 0.05. Δ = mean difference between the second and first measurements; H = Hypothermia; R = mild HIE reference group. Abbreviations: HGB, hemoglobin; MCH, mean corpuscular hemoglobin; MCHC, mean corpuscular hemoglobin concentration; MCV, mean corpuscular volume; RDW, red cell distribution width; PLT, platelet; WBC, white blood cell; NEU, neutrophil; LYM, lymphocyte; MONO, monocyte; BASO, basophil; NRBC, nucleated red blood cell; DNI, delta neutrophil index; LUC, large unstained cells; MPV, mean platelet volume. Statistically significant *p* values are defined as *p* < 0.05.

**Table 3:** RDW and RDW-to-platelet ratio according to HIE severity (Sarnat stages I–III).

Parameter	Stage I (*n* = 66)	Stage II (*n* = 142)	Stage III (*n* = 21)	*p* Value
RDW at birth (%)	17.03 ± 1.35	17.18 ± 1.31	18.05 ± 1.49	0.014
RDW at 72 h (%)	16.46 ± 1.76	16.99 ± 1.71	18.01 ± 1.66	0.009
ΔRDW (72 h–birth, %)	−0.58 ± 1.40	−0.19 ± 1.15	−0.05 ± 0.65	0.287
RPR at birth	0.07 ± 0.03	0.09 ± 0.13	0.16 ± 0.16	<0.001
RPR at 72 h	0.06 ± 0.03	0.11 ± 0.18	0.27 ± 0.30	<0.001
ΔRPR (72 h–birth)	−0.01 ± 0.03	0.02 ± 0.19	0.10 ± 0.35	<0.001

Values are presented as mean ± standard deviation. Δ indicates change between birth and 72 h. RDW: red cell distribution width; RPR: RDW-to-platelet ratio. Continuous variables were compared across Sarnat stages using ANOVA or Kruskal–Wallis tests, as appropriate. Where the overall comparison was statistically significant, post hoc pairwise comparisons were performed with adjustment for multiple testing. For RDW (birth and 72 h), significant differences were observed between Sarnat stage III and stages I and II, whereas the comparison between stages I and II was not significant. For RPR at birth, significant differences were observed between Sarnat stage III and stages I and II, while stages I and II did not differ significantly. For RPR at 72 h, all pairwise comparisons (I vs. II, I vs. III, and II vs. III) were statistically significant. Statistically significant *p* values are defined as *p* < 0.05.

**Table 4:** Exploratory multivariable linear regression analysis for RDW and RPR at 72 h of life.

Predictor	RDW at 72 h (β, 95% CI)	*p* Value	RPR at 72 h (β, 95% CI)	*p* Value
Sarnat Stage II vs. I	0.36 (−0.13 to 0.84)	0.148	0.02 (0.00 to 0.04)	0.043
Sarnat Stage III vs. I	0.50 (−0.16 to 1.16)	0.139	0.09 (−0.09 to 0.27)	0.338
Birth RDW or RPR	0.96 (0.89 to 1.03)	<0.001	0.29 (−0.20 to 0.78)	0.242
Gestational age (weeks)	0.02 (−0.03 to 0.07)	0.392	0.00 (−0.01 to 0.01)	0.641
Male sex	0.23 (−0.12 to 0.57)	0.195	−0.03 (−0.09 to 0.02)	0.190
Early-onset sepsis	0.03 (−0.61 to 0.67)	0.921	−0.04 (−0.09 to 0.01)	0.148
Invasive mechanical ventilation	0.06 (−0.60 to 0.72)	0.855	−0.04 (−0.18 to 0.10)	0.586
Inotropic support	0.06 (−0.63 to 0.76)	0.856	0.13 (−0.05 to 0.31)	0.150

Exploratory multivariable linear regression models evaluating RDW and RPR values at 72 h of life. Birth values were included as covariates to account for baseline differences. Regression coefficients (β) are presented with 95% confidence intervals. Models were adjusted for clinically relevant covariates and used variance-robust estimation. RDW: red cell distribution width; RPR: RDW-to-platelet ratio. Statistically significant *p* values are defined as *p* < 0.05.

### 3.2. Leukocyte Parameters

White blood cell (WBC) and neutrophil (NEU) counts showed significant postnatal declines in both groups (all *p* < 0.001). The magnitude of WBC reduction was greater in the hypothermia group (Δ = −9.75 vs. −6.76, *p* = 0.029), whereas the degree of neutrophil decrease was similar between the groups. Lymphocyte (LYM) counts decreased significantly only in the reference group (*p* = 0.023), with a significant Δ difference between groups (*p* = 0.031). Monocyte (MONO) levels decreased significantly in the hypothermia group (*p* < 0.001) but remained unchanged in the mild HIE reference group, resulting in a significant Δ difference (*p* = 0.006). Basophil (BASO) counts declined significantly in both groups (*p* < 0.001), although the intergroup Δ difference was not statistically significant. Large unstained cells (LUCs) decreased significantly under hypothermia (*p* < 0.001) but remained stable in infants in the mild HIE reference group, with a significant Δ difference (*p* = 0.027). The delta neutrophil index (DNI) did not significantly change in either group.

### 3.3. Platelet Parameters

Platelet (PLT) counts remained stable in the hypothermia group (*p* = 0.830) but increased significantly in the reference group (*p* = 0.004). The Δ difference between the groups was statistically significant (*p* = 0.007), suggesting that platelet recovery may be delayed in cooled infants. The mean platelet volume (MPV) did not differ significantly between the groups or across time points.

## 4. Discussion

In this study, we evaluated the patterns of RDW and the RDW-to-platelet ratio across the clinical spectrum of hypoxic–ischemic encephalopathy under standard-of-care treatment. Although several studies have explored hematologic changes during therapeutic hypothermia, data specifically focusing on RDW and RPR remain limited. The demographic and clinical characteristics of our cohort were consistent with those of previous reports on hypoxic–ischemic encephalopathy. Differences in gestational age, Apgar scores, and cord blood parameters reflect the expected clinical severity in infants requiring therapeutic hypothermia. Variations in inborn status and the need for respiratory or inotropic support were also in line with earlier findings, indicating that infants managed with cooling typically present with more pronounced perinatal compromise. Other perinatal and maternal characteristics, including birth weight, gestational diabetes, and hypertensive disorders, showed distributions similar to those described in the literature, supporting the representativeness of the study population. Accordingly, the observed findings should be interpreted as differences in early hematologic trajectories across HIE severity under standard-of-care treatment rather than isolated causal effects attributable solely to therapeutic hypothermia.

The initial RDW values in infants diagnosed with HIE were higher than those reported for healthy newborns, which may reflect the inflammatory response and tissue injury that follow perinatal asphyxia. Tonbul et al. reported a mean RDW level of 16.2 ± 1.3 in healthy term neonates [11]. In our cohort, RDW values did not significantly change after therapeutic hypothermia and remained largely stable before and after cooling. Although RDW did not show intragroup significance within the hypothermia cohort, the intergroup Δ demonstrated a blunted decline relative to the reference group. In contrast, RPR tended to increase slightly during hypothermia. This trend may reflect both the inflammatory burden associated with HIE and thrombocytopenia commonly encountered during cooling. Previous studies have demonstrated that both perinatal asphyxia and hypothermia considerably increase the risk of thrombocytopenia in newborns [12]. Taken together, our data suggest that the 72 h cooling period neither normalizes RDW nor prevents a mild elevation in RPR, which may be secondary to a decline in platelets.

The relatively high and stable RDW values observed in our cohort may be associated with inflammation and oxidative stress triggered by HIE. Hypoxic–ischemic injury leads to cytokine release and early egress of immature erythrocytes into the circulation, resulting in anisocytosis. RDW, a quantitative indicator of erythrocyte size variability, was initially used to differentiate anemia etiologies but is now recognized as a marker of oxidative stress and inflammation in various chronic inflammatory conditions, such as heart failure, COPD, and pulmonary hypertension [13]. Similarly, the intense inflammatory burden of perinatal asphyxia may have contributed to the elevated RDW. Although therapeutic hypothermia aims to mitigate neurological injury by reducing metabolic demand and has been reported to attenuate oxidative stress [14], erythrocyte turnover kinetics are slow. Therefore, a limited 72 h cooling period is not expected to produce rapid reductions in RDW. Furthermore, the decline in platelet counts frequently seen under cooling may also contribute to the observed increase in RPR, consistent with earlier reports highlighting the association between hypothermia and thrombocytopenia in asphyxiated newborns [12]. Thus, an elevated RDW likely reflects the underlying hypoxic and inflammatory processes in HIE, with minimal acute reversibility during short-term hypothermia.

Although data on RDW and RPR in the context of HIE are limited, comparisons with other neonatal conditions may provide valuable insights [15,16,17]. In neonatal sepsis, RDW, and particularly RPR, increase markedly as indicators of systemic inflammation and have been proposed as auxiliary biomarkers for diagnosis and prognosis [6]. Similarly, premature infants who develop bronchopulmonary dysplasia exhibit significantly elevated RDW levels from the first week of life, consistent with chronic lung inflammation and oxidative stress [13]. Elevated RDW values have also been described in pediatric and adult pulmonary hypertension and may correlate with disease severity [13]. These findings suggest that an increased RDW reflects underlying inflammation, tissue hypoxia, and organ dysfunction. The relatively higher RDW and mild increase in RPR observed in newborns with HIE may also reflect the inflammatory burden and disease severity. Accordingly, RDW and related hematologic indices may serve as useful adjunctive biomarkers for understanding the pathophysiological processes of HIE.

Beyond RDW and RPR, several additional hematologic parameters demonstrated expected trends in response to perinatal asphyxia and therapeutic hypothermia. White blood cell (WBC) and neutrophil counts decreased significantly in both groups, consistent with the literature describing bone marrow suppression and leukocyte redistribution during cooling. Lymphocyte counts decreased significantly only in the reference group, yielding a significant Δ difference. Transient declines in lymphocytes and monocytes may reflect stress-related leukocyte shifts, which have also been documented in cooled neonates. Platelet counts remained stable or slightly decreased in the hypothermia group, consistent with previous findings of mild, self-limited thrombocytopenia during cooling therapy. Overall, these observations align with those of existing literature. This supports the interpretation that early inflammatory and hematopoietic trajectories differ between infants treated with standard-of-care hypothermia and those in the mild HIE reference group without causing clinically significant cytopenia [8,9,18]. Consistent with these findings, exploratory severity-based analyses suggested that higher RPR levels were associated with increasing HIE severity, supporting the notion that early platelet-related hematologic responses may reflect the extent of hypoxic–ischemic injury rather than treatment effects alone. Given the clinical indications for therapeutic hypothermia, the hematological changes observed in this study are more likely to reflect the severity of hypoxic–ischemic encephalopathy rather than a causal effect of hypothermia itself.

### 4.1. Strengths

This study provides a detailed descriptive analysis of early RDW and RDW-to-platelet ratio trajectories across the spectrum of hypoxic–ischemic encephalopathy using routinely available hematologic parameters. The inclusion of infants with mild HIE as a clinically relevant reference group allowed the interpretation of hematologic patterns within ethical and real-world clinical constraints.

### 4.2. Limitations

This study has several limitations—the retrospective, single-center design limits causal inference and generalizability. Infants with mild HIE do not represent a physiological control group, and residual confounding related to baseline disease severity cannot be excluded. RDW and RPR were assessed only within the first 72 h of life, and long-term hematologic, neurological, or neurodevelopmental outcomes were not evaluated. In addition, therapeutic interventions administered during cooling may have influenced the hematologic indices. Therefore, these findings should be interpreted as descriptive rather than prognostic. The exploratory nature of the multivariable analyses and the limited number of infants with severe HIE warrant cautious interpretation of regression-based findings.

## 5. Conclusions

Therapeutic hypothermia remains the standard of care for improving survival and neurological outcomes in newborns with hypoxic–ischemic encephalopathy. In this study, hypothermia did not exert an immediate normalizing effect on RDW or RPR, and elevated values during the acute period likely reflected the underlying hypoxic injury and systemic inflammatory response rather than a reversible hematologic effect. RDW-based indices, as simple and widely accessible biomarkers, may provide complementary information about early inflammatory and hematopoietic dynamics in HIE and may be useful adjunctive parameters during clinical monitoring. Although these markers should not be used in isolation to guide treatment decisions, they may provide complementary descriptive information on early hematologic trajectories, but should not be used for risk stratification or to guide clinical decisions in isolation. These hematologic changes should be interpreted as expected physiological responses during HIE and therapeutic hypothermia and should not prompt unnecessary clinical interventions when considered in isolation. Further prospective multicenter studies are warranted to clarify the long-term prognostic implications of RDW and RPR and determine their potential roles in predicting neurodevelopmental outcomes.

## Figures and Tables

**Table 1:** Baseline clinical characteristics of neonates with and without hypothermia treatment.

Parameter	Hypothermia (*n* = 166)	Mild HIE Reference Group (*n* = 63)	*p* Value
GA (weeks)	38.32 ± 2.81	39.00 ± 1.58	0.038
Vaginal delivery, *n* (%)	73 (44.0%)	30 (47.6%)	0.729
Birth weight (g)	3109.13 ± 501.62	3207.65 ± 424.88	0.142
Birth weight percentile	2.99 ± 0.42	3.00 ± 0.36	0.837
Apgar 1 min	4 (0–7)	6 (2–8)	<0.001
Apgar 5 min	7 (0–9)	8 (5–9)	<0.001
Cord pH	6.93 ± 0.15	7.04 ± 0.09	<0.001
Cord base excess (mmol/L)	−17 (−39–−9)	−14 (−23–−10)	0.001
Cord lactate (mmol/L)	10.02 ± 4.56	7.29 ± 3.01	0.003
IMV days	0 (0–27)	0 (0–1)	<0.001
Inborn, *n* (%)	59 (35.5%)	55 (87.3%)	<0.001
Female, *n* (%)	77 (46.4%)	25 (39.7%)	0.446
IVF, *n* (%)	0 (0.0%)	1 (1.6%)	0.619
GDM, *n* (%)	7 (4.3%)	3 (4.8%)	1.000
Chronic hypertension, *n* (%)	2 (1.2%)	0 (0.0%)	0.930
Preeclampsia, *n* (%)	5 (3.1%)	2 (3.2%)	1.000
IUGR, *n* (%)	4 (2.4%)	0 (0.0%)	0.492
PROM/PPROM, *n* (%)	7 (4.3%)	1 (1.6%)	0.563
Early-onset sepsis, *n* (%)	22 (13.3%)	6 (9.5%)	0.577
Late-onset sepsis, *n* (%)	42 (25.5%)	2 (3.2%)	<0.001
Seizures, *n* (%)	59 (36.0%)	0 (0.0%)	–
Inotrope support, *n* (%)	95 (58.6%)	0 (0.0%)	–
Oliguria, *n* (%)	51 (32.7%)	0 (0.0%)	–
Discharged, *n* (%)	151 (91.0%)	60 (95.2%)	0.068

Values are expressed as mean ± SD, median (min–max), or *n* (%), as appropriate. Statistical comparisons between groups were performed using the independent *t*-test, Mann–Whitney U test, or chi-square test, depending on the data distribution. Abbreviations: GA, gestational age; IMV, invasive mechanical ventilation; IVF, in vitro fertilization; GDM, gestational diabetes mellitus; Statistically significant *p* values are defined as *p* < 0.05.

## Data Availability

The data presented in this study are available upon reasonable request from the corresponding author. The data are not publicly available due to privacy and ethical reasons.

## Supplementary Materials

The following supporting information can be downloaded at: https://www.mdpi.com/article/10.3390/children13010100/s1, Table S1. Exploratory Multivariable Linear Regression Analysis for Changes in RDW and RPR (Entire Cohort). Table S2. Exploratory Multivariable Linear Regression Analysis in Infants Receiving Therapeutic Hypothermia Only (Sarnat Stages II–III).

## Abbreviations

The following abbreviations are used in this manuscript: HIE, hypoxic–ischemic encephalopathy; RDW, red cell distribution width; RPR, RDW-to-platelet ratio; HGB, hemoglobin; MCV, mean corpuscular volume; MCH, mean corpuscular hemoglobin; MCHC, mean corpuscular hemoglobin concentration; WBC, white blood cell; NEU, neutrophil; LYM, lymphocyte; MONO, monocyte; BASO, basophil; NRBC, nucleated red blood cell; DNI, delta neutrophil index; LUC, large unstained cells; PLT, platelet; MPV, mean platelet volume; EOS, early-onset sepsis; LOS, late-onset sepsis.
