# Supplementary material for: Red Cell Distribution Width and RDW-to-Platelet Ratio Patterns Across the Spectrum of Hypoxic–Ischemic Encephalopathy
Source: Children (Basel). 2026 Jan 10;13(1):100. doi: 10.3390/children13010100 (PMC12840257; doi:10.3390/children13010100)
Supplement: Supplementary file 1 [file children-13-00100-s001.zip › children-4044632-supplementary.pdf]

Supplementary Table S1. Exploratory Multivariable Linear Regression Analysis for Changes in RDW and RPR (Entire Cohort)

Predictor	ΔRDW (β, 95% CI)	p value	ΔRPR (β, 95% CI)	p value
**Sarnat Stage II vs I**	0.36 (−0.13 to 0.85)	0.148	0.01 (−0.02 to 0.04)	0.335
**Sarnat Stage III vs I**	0.48 (−0.17 to 1.13)	0.150	0.05 (−0.14 to 0.23)	0.638
**Gestational age (weeks)**	0.03 (−0.02 to 0.07)	0.313	0.00 (−0.01 to 0.02)	0.660
**Male sex**	0.21 (−0.13 to 0.55)	0.225	−0.04 (−0.09 to 0.02)	0.205
**Early-onset sepsis**	0.02 (−0.63 to 0.68)	0.944	−0.02 (−0.07 to 0.04)	0.550
**Invasive mechanical ventilation**	0.11 (−0.54 to 0.76)	0.741	−0.03 (−0.15 to 0.09)	0.602
**Inotropic support**	−0.03 (−0.70 to 0.64)	0.926	0.10 (−0.06 to 0.27)	0.226

Exploratory linear regression models evaluating changes (Δ) in RDW and RPR between birth and 72 h. Regression coefficients (β) are presented with 95% confidence intervals. Models were adjusted for clinically relevant covariates. Variance-robust standard errors were applied.

Supplementary Table S2. Exploratory Multivariable Linear Regression Analysis in Infants Receiving Therapeutic Hypothermia Only (Sarnat Stages II–III)

Predictor	ΔRDW (β, 95% CI)	p value	ΔRPR (β, 95% CI)	p value
**Sarnat Stage III vs II**	0.13 (−0.44 to 0.71)	0.650	0.01 (−0.07 to 0.09)	0.792
**Gestational age (weeks)**	0.00 (−0.04 to 0.04)	0.955	0.00 (−0.01 to 0.01)	0.997
**Male sex**	0.04 (−0.26 to 0.35)	0.779	−0.01 (−0.03 to 0.02)	0.565

**Early-onset sepsis**	0.15 (−0.38 to 0.68)	0.573	−0.01 (−0.03 to 0.02)	0.630
**Invasive mechanical ventilation**	−0.07 (−0.63 to 0.50)	0.821	−0.02 (−0.09 to 0.06)	0.686
**Inotropic support**	0.17 (−0.31 to 0.65)	0.479	0.04 (−0.04 to 0.13)	0.333

Exploratory regression models restricted to infants treated with therapeutic hypothermia. Due to the limited number of infants with Sarnat Stage III, confidence intervals are wide and results should be interpreted cautiously.
